# 

**Published:** 1907-03

**Authors:** 


### The American Journal of Dental Science

The Official Organ of the Florida, Georgia and South Carolina State Dental Societies

**Third Series**

VOL. XXXVIII MARCH, 1907 NO. 3

EDITORS

WM. GIRD BEECROFT, D. D. S., Madison. Wis. B. H. TEAGUE, D. D. S., Aiken, S. C.

Published on the 10th day of each month.
Subscription price $1.00 per year.
ENTERED AS SECOND-CLASS MATTER, MADISON, WIS.

Address all communications, both business and editorial, to
**The Dental Publishing Company, Madison, Wisconsin**

### MEETINGS.

Georgia State Dental Society meets at Atlanta, May 7, 8, 9.

Nebraska State Dental Society meets at Lincoln, May 21, 22, 23.

Kentucky State Dental Association meets at Louisville, May 20, 21, 22.

Mississippi Dental Association meets at Meridian, May 28, 29, 30.

Tennessee State Dental Association meets at Knoxville, July 9, 10, 11.

National Association of Dental Faculties meets in Minneapolis, commencing at 2 P, M. Friday July 26-th. The Executive Committe will meet at 10 A. M. the same day. The West Hotel has been selected as headquarters.

---

### JAMESTOWN EXPOSITION, NORFOLK, VA., 1907.

THE JAMESTOWN DENTAL CONVENTION, NORFOLK, VIRGINIA,

September 10-12th, 1907.

Committee on Organization. Burton Lee Thorpe, Chairman; St, Louis, Mo; H. Wood Campbell, Secretary, Suffolk, Va; R. H. Walker, Norfolk, Va; F. W. Stiff, Treasurer; Thos. P. Hinman, Atlanta, Ga; J. E. Chace, Ocalo, Fla; Clarence J. Grieves, Baltimore, Md.

The Jamestown Dental Convention will be held in an especially equipped building on the Exposition Grounds, which was built for the purpose of accomodating this convention, the building is equipped with an auditorum, committee rooms, and excellent facilities for conducting dental clinics and for holding exhibits. All of these will be held in this building. The entrance to the building is outside of the grounds, however, one may obtain access to the grounds through it. The building is wired with both direct and alternating current, equipped with running water, well lighted and contains all modern conveniences, thus making it an ideal conventional hall. The exhibits are under the management of Dr. John W. Manning, Bank of Commerce Bldg., Norfolk, Virginia. To him exhibitors should apply at once for space. Price per foot and a plat of the hall will be sent upon request. The clinics are under supervision and idrect control of Dr. C. J. Grieves, Park & Madison Aves., Baltimore, Md. His assistants are, Drs. Baskerville Bridgeforth, Richmond, Virginia, E. J. Tucker, Roxboro, N.C. Herbert Johnson, Macon, Ga., F. A. Bowles. Washington, D. C. and Joseph T. Meadors, Nashville, Tenn. The prospects are that the Jamestown clinic will be the largest and most complete dental clinic ever held. Assistants clinic chairman have been appointed in each state in the Union and nearby countries, viz; Canada, Mexico, Cuba and Hawaii. From these come reports of the enlistment of the best clinic talent in their respectve states and countries.

Membership chairmen have been appointed in the various states and countries. The membership committee is headed by Dr. F. W. Stiff, the general chairman, 600 East Grace St., Richmond, Va., who already reports memberships rapidly coming in. The Hotel head-quarters will be at the Inside Inn, where reasonable rates and excellent accomodations are assured. The Inside Inn generously offers numerous halls and committee rooms free of charge to the various college fraternities and alumni, who are invited to hold their meetings in these rooms. Later reports as to hotel accomodations and prices will appear in a subsequent issue of this journal. The Membership fee is five dollars, which will entitle members to a bound copy of the proceedings. A half rate $2.50 is made to bona fidi dental students upon certificates from the Deans of their colleges, and when presented to the state chairman of the membership committee, for endorsement and acceptance, will entitle them to the rights and privileges of the convention. The essayists are to be, Prof. W. D. Miller, of Berlin, Germany, Dr. F. T. Van Woert, Brooklyn, N. Y. whose subject is, "Is the cement filling the filling of the future"? Dr. Chas. L. Alexander, of Charlotte, N. C. who will present a paper on "Gold Inlay". The other assayists will be announced later. Dr E. P. Beadles was elected by the Committee on Organization, in Feb. to go to Europe and extend a cordial invitation to the dental socities and individual dentists to attend the Convention.

